# Low hemoglobin is associated with postoperative cerebral infarction in moyamoya disease: development of a predictive model based on low hemoglobin

**DOI:** 10.3389/fneur.2024.1489430

**Published:** 2025-01-07

**Authors:** Haitao Wu, Tingxuan Wang, Fangbao Li, Yue Bao, Bin Lu, Luo Li

**Affiliations:** ^1^Qingdao University Medical College, Qingdao University, Qingdao, China; ^2^Dalian Medical University, Dalian, China; ^3^Department of Neurosurgery, University of Health and Rehabilitation Sciences (Qingdao Municipal Hospital), Qingdao, China

**Keywords:** moyamoya disease, hemoglobin, revascularization, postoperative cerebral infarction, prediction model

## Abstract

**Background:**

Anemia is considered a risk factor for cardiovascular disease. However, there is little evidence regarding the relationship between hemoglobin (HB) and cerebral infarction after revascularization in patients with moyamoya disease (MMD). This study aimed to explore the relationship between postoperative cerebral infarction and HB in patients with MMD and to establish a predictive model.

**Methods:**

Demographic information and different HB levels (the preoperative and postoperative HB, highest and lowest HB, and mean HB during hospitalization) of 112 patients with MMD were collected, of which 11 had cerebral infarction after revascularization.

**Results:**

In the binomial logistic regression analysis, low HB levels were an independent risk factor for cerebral infarction after revascularization, which also led to a worse long-term prognosis in patients with MMD. The risk factors, including Pre-HB, Post-HB, type of MMD, and hypertension (HTN), were incorporated into the receiver operating characteristic curve, which yielded an area under the curve (AUC) of 0.83.

**Conclusion:**

The prediction model was visualized using a nomogram, and a clinical decision curve was drawn to evaluate the net benefit of clinical decisions.

## 1 Introduction

Moyamoya disease (MMD) is a rare, chronic, progressive cerebrovascular disease that mainly affects the distal ends of the bilateral internal carotid artery (1). It often involves the proximal end of the anterior and middle cerebral arteries and is accompanied by abnormal neovascularization at the skull base (2). Moyamoya disease is named for its distinctive appearance on cerebral angiography, where the abnormal small blood vessels form a pattern resembling a hazy cloud of smoke (3). Epidemiology shows that the incidence of MMD in Asian populations is approximately 6 cases per 100,000 people (4), and a review from the United States showed that there were only 0.086 cases of MMD per 100,000 people (5). Peak incidence occurs in two age groups: children aged approximately 5 years and adults aged 40 years (6, 7). MMD is divided into ischemic and hemorrhagic types, with the common symptoms being mainly hemiplegia, dysarthria, aphasia, and cognitive impairment (8); common sites of hemorrhagic types are the ventricles, basal ganglia, and subarachnoid space (9, 10). There is currently no cure for diseased blood vessels in the brain. Revascularization surgery to redirect blood flow to the diseased hemisphere and reduce the risk of ischemic stroke and possible cognitive impairment is the mainstay of treatment (8, 11, 12). Revascularization surgery includes direct, indirect, and combined surgeries. The improvement of blood flow in the diseased cerebral hemisphere is obvious with revascularization surgery, but the ensuing postoperative complications [intracranial hemorrhage, ischemic stroke, impaired neurological function, epilepsy, and hyperperfusion syndromes (4, 11, 13)] may lead to a potential deterioration of neurological function or even permanent neurological loss (14, 15). Therefore, the timely and accurate assessment and prevention of postoperative complications in patients with MMD is one of the current priorities for clinical treatment.

Studies have shown that low hemoglobin (HB) levels after cerebral hemorrhage are not only associated with hematoma expansion (16, 17) but also with higher mortality and an increased risk of adverse outcomes (18–20). Additionally, anemia is a risk factor for chronic heart failure (21) and myocardial infarction (22). However, there is a lack of literature on the effects of HB on postoperative cerebral infarction in patients with MMD. This study aimed to evaluate the effect of HB on cerebral ischemic complications after revascularization and establish a predictive model.

## 2 Methods

### 2.1 Patients

Electronic hospital files of patients with MMD admitted to Qingdao Municipal Hospital for revascularization between January 2012 and March 2023 were retrospectively screened. Patients were only considered for further analysis if they met all the following eligibility criteria: (1) diagnosed with MMD through digital subtraction angiography (DSA) or MR angiography, according to the guidelines published by the MMD Research Committee of Japan (23). (2) No relevant contraindications to surgery or those who had already undergone surgical revascularization. (3) Aged >18 years, with a history of brain tumors, cranial irradiation, Down syndrome, neurofibromatosis, meningitis, or sickle cell disease. All procedures were performed according to the guidelines of the Declaration of Helsinki.

Patient demographics including sex, age, hypertension (HTN), diabetes mellitus (DM), coronary artery disease (CHD), smoking history, admission modified Rankin scale (mRS) score, >1 year after surgery mRS score, type of symptoms, surgical type, and imaging characteristics were collected. Suzuki staging, determined by imaging, was evaluated by at least two neurosurgeons. All HB concentrations measured during hospitalization were extracted from the database. There were multiple measurements of HB concentrations during each patient’s hospitalization, and to minimize errors, the last time in patients with preoperative HB concentrations (Pre-HB), the first time in patients with postoperative HB concentrations (Post-HB), mean HB concentrations (the sum/number of all HB that could be recorded during the patient’s hospital stay), and lowest and highest HB concentrations during hospitalization were documented.

### 2.2 Postoperative cerebral infarction

A radiologist with a senior professional title and a neurosurgeon with a senior professional title jointly confirmed postoperative cerebral infarction according to the typical symptoms and imaging findings (such as computed tomography [CT] and CT perfusion diffusion-weighted imaging) of patients with MMD.

### 2.3 Statistical analysis

IBM SPSS Statistics 27.0 and R 4.2 statistical software were used for all statistical analyses. Participants were divided into two groups according to the occurrence of postoperative cerebral infarction. Categorical variables were presented as frequencies, and differences were compared using the chi-squared test or Fisher’s exact test. Based on the Kolmogorov–Smirnov test results, normally distributed continuous variables were presented as mean ± standard deviation, and comparisons were performed using the t-test. Non-normally distributed continuous variables were presented as median and interquartile range (IQR), with differences between groups tested using the Mann–Whitney U-test. All HB and potential risk factors were included in univariate and multifactorial binomial logistic regression analyses. We use restricted cubic splines to flexibly model and visualize the relationship between risk factors and postoperative cerebral infarction. The receiver operating characteristic curve (ROC) and area under the curve (AUC) were used as the indices of distinguishing ability to construct a prediction model. The 1,000-repetition bootstrap resampling strategy was performed for optimistic correction, and calibration was confirmed by the Hosmer–Lemeshow test. To better apply the new risk factors to the clinical study, a nomogram prediction model should be established. The decision curve analysis (DCA) curve provides a comprehensive assessment of postoperative cerebral infarction of MMD. All statistical tests were two-tailed, and a *p*-value of <0.05 was considered statistically significant.

## 3 Results

**Figure 1:**
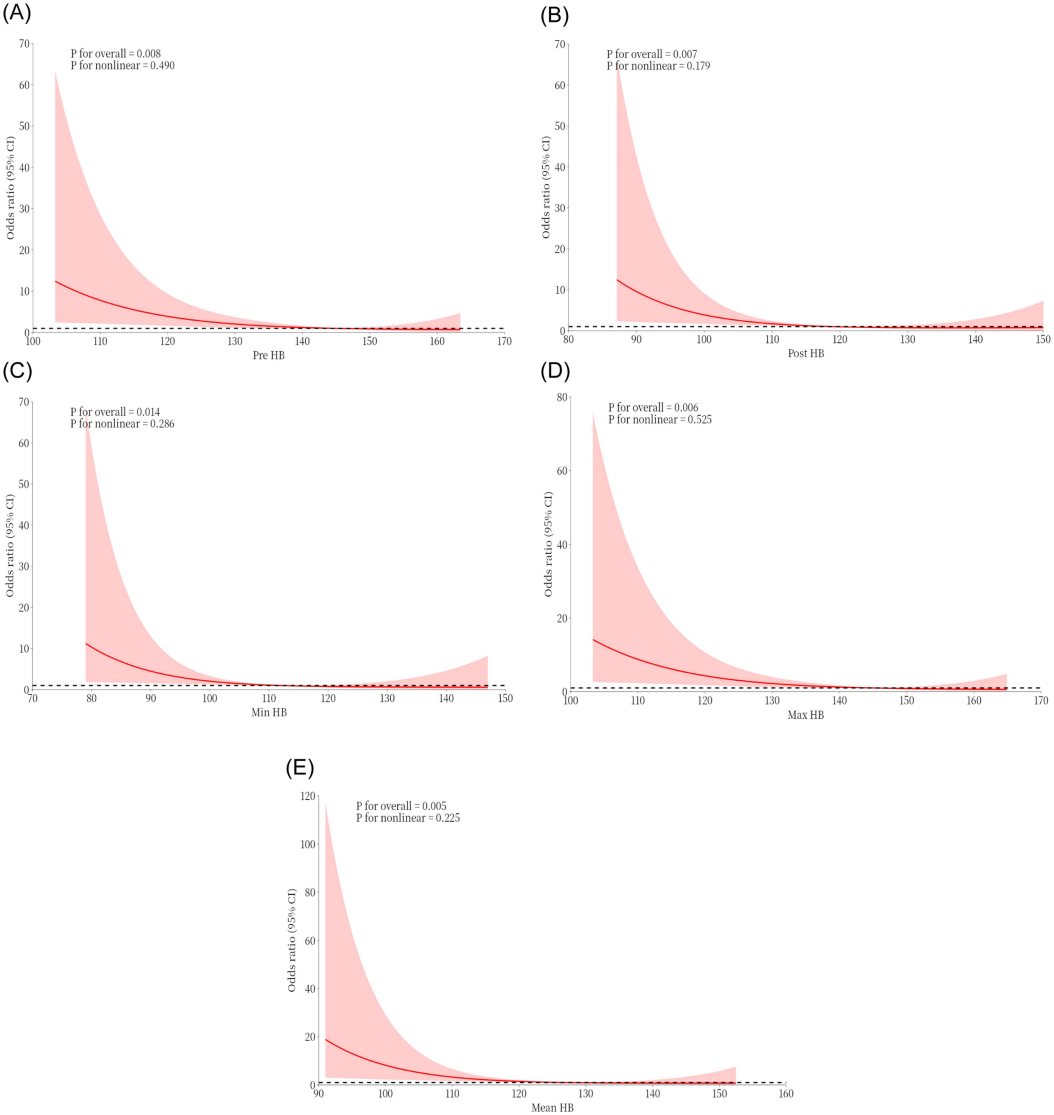
Restricted cubic spline plots were used to visualize the effects of Pre-HB **(A)**, Post-HB **(B)**, Min HB **(C)**, Max HB **(D)**, and Mean HB **(E)** at different levels on postoperative cerebral infarction of moyamoya disease.

Following the inclusion and exclusion criteria, a total of 112 patients were included in this study. Table 1 summarizes the basic clinical data of patients with MMD. The mean age of the patients on admission was 48.08 ± 11.68 years, and there were more male patients (55.36%). The Pre-HB concentration of the patients was 138.82 ± 19.19 g/L, the Post-HB concentration was 119.40 ± 19.35 g/L, the mean HB concentration during hospitalization was 124.61 ± 18.01 g/L, the Min HB concentration during hospitalization was 113.31 ± 19.44 g/L, and the Max HB concentration during hospitalization was 139.19 ± 19.28 g/L. The MMD type of 93 patients (83.04%) was cerebral ischemia, and the MMD type of 19 patients (16.96%) was cerebral hemorrhage. The Suzuki stage was I in 1 patient (0.89%), II in 33 patients (29.46%), III in 53 patients (47.32%), IV in 19 patients (16.96%), V in 6 patients (5.36%), and none were stage VI. A total of 112 revascularization procedures were performed, including 65 (58.04%) direct bypass procedures, 22 (19.64%) indirect bypass procedures, and 25 (22.32%) combined procedures. Eleven cases (9.8%) of MMD patients had postoperative cerebral infarction, of which four cases (36.36%) occurred after direct bypass surgery, three cases (27.27%) after indirect bypass surgery, and four cases (36.36%) after combined surgery. In patients with postoperative cerebral infarction, patients with HTN (63.64% > 48.15%), hemorrhagic (36.36% > 14.85%), high mRS (63.64% > 28.71%), mRS Score was 3–6 points (63.64% > 28.71%), >1 year after surgery mRS score >3–6 points (54.55% > 45.45%) is higher than patients who did not have a postoperative cerebral infarction. At the same time, Pre-HB (123.73 ± 26.65 g/L < 140.47 ± 17.61 g/L), Post-HB (104.55 ± 24.09 g/L < 121.02 ± 18.18 g/L), Max HB (123.73 ± 26.65 g/L < 140.87 ± 17.67 g/L), Min HB (99.36 + 21.96 g/L < 114.83 + 18.64 g/L), and Mean HB (109.91 ± 23.99 g/L < 126.21 ± 16.62 g/L) were lower in patients who experienced postoperative cerebral infarction than those who did not. We used a restricted cubic spline to visualize HB and postoperative cerebral infarction in patients with MMD. Because there are inherent differences in HB values between men and women, we set sex as a covariate to reduce the effect of gender on the results. The results show that when the Pre-HB > 113.2 g/L (Figure 1A), Post-HB > 94.1 g/L (Figure 1B), Min HB is >86.2 g/L (Figure 1C), Max HB > 115 g/L (Figure 1D), and Mean HB > 102.2 g/L (Figure 1E), the risk of postoperative cerebral infarction was low.

**Table 1:** Comparison between the clinical data of patients with MMD and univariate binary logistic regression results.

Variables	Total (*n* = 112)	Postoperative ischemic complication		*p*- value
		Absent (*n* = 101)	Present (*n* = 11)	
Age	48.08 ± 11.68	47.64 ± 11.57	52.09 ± 12.52	0.234
Sex, *n* (%)				
Male	62 (55.36)	56 (55.45)	6 (54.55)	
Female	50 (44.64)	45 (44.55)	5 (45.45)	0.234
Surgical side, *n* (%)				
Right	62 (55.36)	57 (56.44)	5 (45.45)	
Left	50 (44.64)	44 (43.56)	6 (54.55)	0.234
Disease involved, *n* (%)				
Unilateral	47 (41.96)	42 (41.58)	5 (45.45)	
Bilateral	65 (58.04)	59 (58.42)	6 (54.55)	0.234
Primary type, *n* (%)				
Hemorrhagic	19 (16.96)	15 (14.85)	4 (36.36)	
Ischemic	93 (83.04)	86 (85.15)	7 (63.64)	0.084
Suzuki stage, *n* (%)				
I	1 (0.89)	1 (0.99)	0 (0.00)	
II	33 (29.46)	30 (29.70)	3 (27.27)	0.993
III	53 (47.32)	48 (47.52)	5 (45.45)	0.993
IV	19 (16.96)	17 (16.83)	2 (18.18)	0.993
V	6 (5.36)	5 (4.95)	1 (9.09)	0.992
VI	0 (0)	0 (0)	0 (0)	
Surgical type, *n* (%)				
Direct bypass	65 (58.04)	61 (60.40)	4 (36.36)	
Indirect bypass	22 (19.64)	19 (18.81)	3 (27.27)	0.277
Combined	25 (22.32)	21 (20.79)	4 (36.36)	0.156
Hypertension, *n* (%)				
No	56 (50.00)	52 (51.49)	4 (36.36)	
Yes	56 (50.00)	49 (48.51)	7 (63.64)	0.347
Diabetes, *n* (%)				
No	93 (83.04)	85 (84.16)	8 (72.73)	
Yes	19 (16.96)	16 (15.84)	3 (27.27)	0.345
Coronary heart disease, *n* (%)				
No	105 (93.75)	94 (93.07)	11 (100.00)	
Yes	7 (6.25)	7 (6.93)	0 (0.00)	0.992
Smoking, *n* (%)				
No	80 (71.43)	71 (70.30)	9 (81.82)	
Yes	32 (28.57)	30 (29.70)	2 (18.18)	0.428
Admission mRS score, *n* (%)				
0	1 (0.89)	1 (0.99)	0 (0.00)	
1	34 (30.36)	32 (31.68)	2 (18.18)	0.993
2	41 (36.61)	39 (38.61)	2 (18.18)	0.993
3	21 (18.75)	18 (17.82)	3 (27.27)	0.992
4	12 (10.71)	9 (8.91)	3 (27.27)	0.992
5	3 (2.68)	2 (1.98)	1 (9.09)	0.992
Admission mRS score, *n* (%)				
0–2	76 (67.86)	72 (71.29)	4 (36.36)	
3–6	36 (32.14)	29 (28.71)	7 (63.64)	**0.027**
>1 year after surgery mRS score, *n* (%)				
0–2	84 (75.00)	79 (78.22)	5 (45.45)	
3–6	28 (25.00)	22 (21.78)	6 (54.55)	**0.025**
Pre-HB(g/L)	138.82 ± 19.19	140.47 ± 17.61	123.73 ± 26.65	**0.010**
Post-HB(g/L)	119.40 ± 19.35	121.02 ± 18.18	104.55 ± 24.09	**0.011**
Max HB(g/L)	139.19 ± 19.28	140.87 ± 17.67	123.73 ± 26.65	**0.009**
Min HB(g/L)	113.31 ± 19.44	114.83 ± 18.64	99.36 ± 21.96	**0.016**
Mean HB(g/L)	124.61 ± 18.01	126.21 ± 16.62	109.91 ± 23.99	**0.007**

The bold values indicates that the *p* value is less than 0.05.

**Figure 2:**
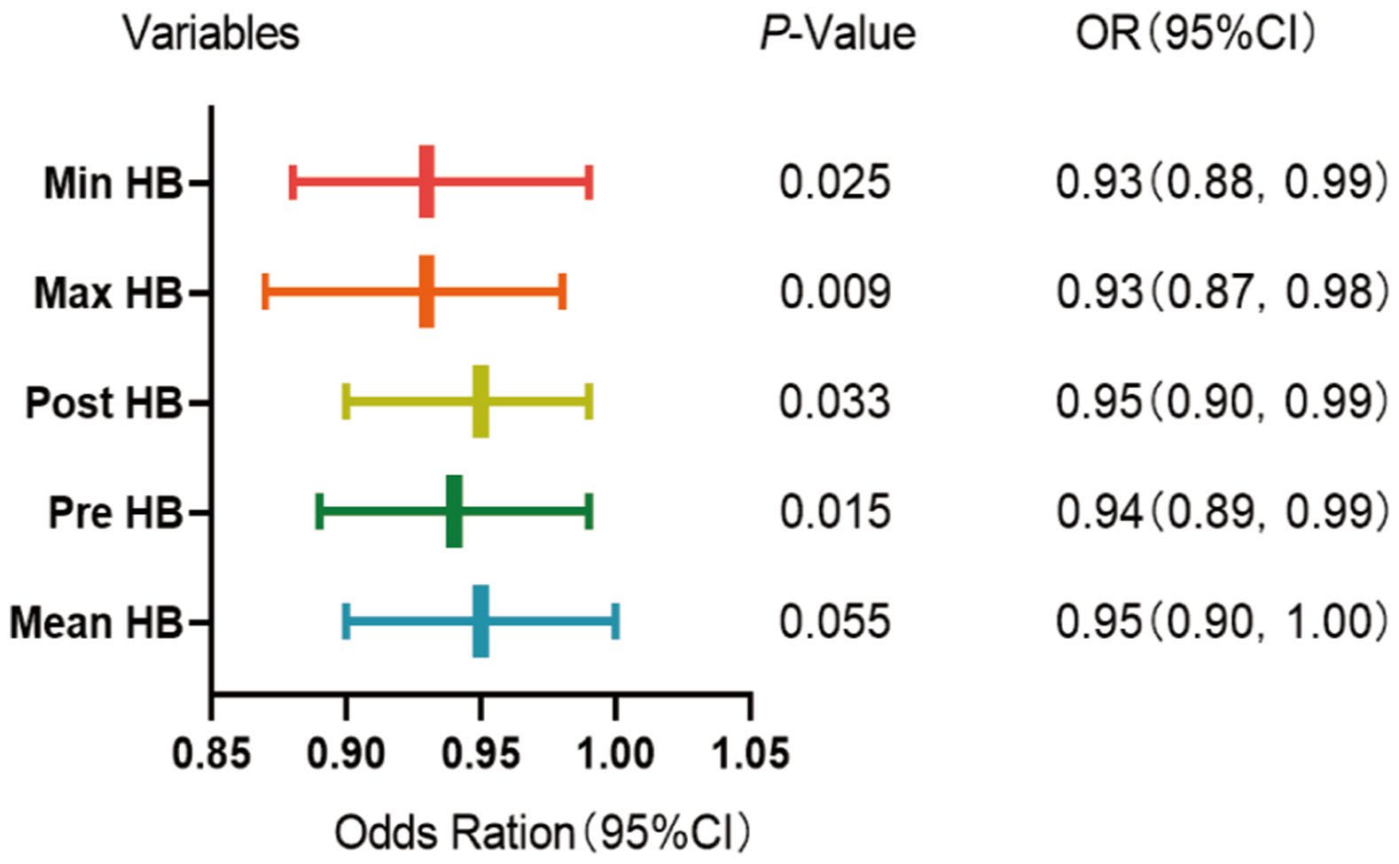
Multivariate binomial logical analysis results between different HB and postoperative cerebral infarction of MMD.

Binary logistic regression analysis was used to explore the risk factors for postoperative cerebral infarction in patients with MMD. Univariate binary logistic regression showed (Table 1) that high mRS scores at admission (*p* = 0.027), Pre-HB (*p* = 0.010), Post-HB (*p* = 0.011), maximum HB (*p* = 0.009), minimum HB (*p* = 0.016), and Mean HB (*p* = 0.007) were risk factors for postoperative cerebral infarction. Because of the collinearity between different HB measurements in the same patient, each time we introduced only one HB value for multivariate analysis with all other variables. The results showed (Figure 2) that Pre-HB (*p* = 0.015; OR = 0.94, 95% CI = 0.89, 0.99), Post-HB (*p* = 0.033; OR = 0.95, 95% CI = 0.90, 0.99), the Max HB (*p* = 0.009; OR = 0.93, 95% CI = 0.87, 0.98), and Min HB (*p* = 0.025; OR = 0.93, 95%CI = 0.88, 0.99) were still independent risk factors for postoperative cerebral infarction, while Mean HB (*p* = 0.055; OR = 0.95, 95%CI = 0.90,1.00) were not independent risk factors for postoperative cerebral infarction of MMD.

### 3.1 Development of a predictive model for postoperative cerebral infarction

**Figure 3:**
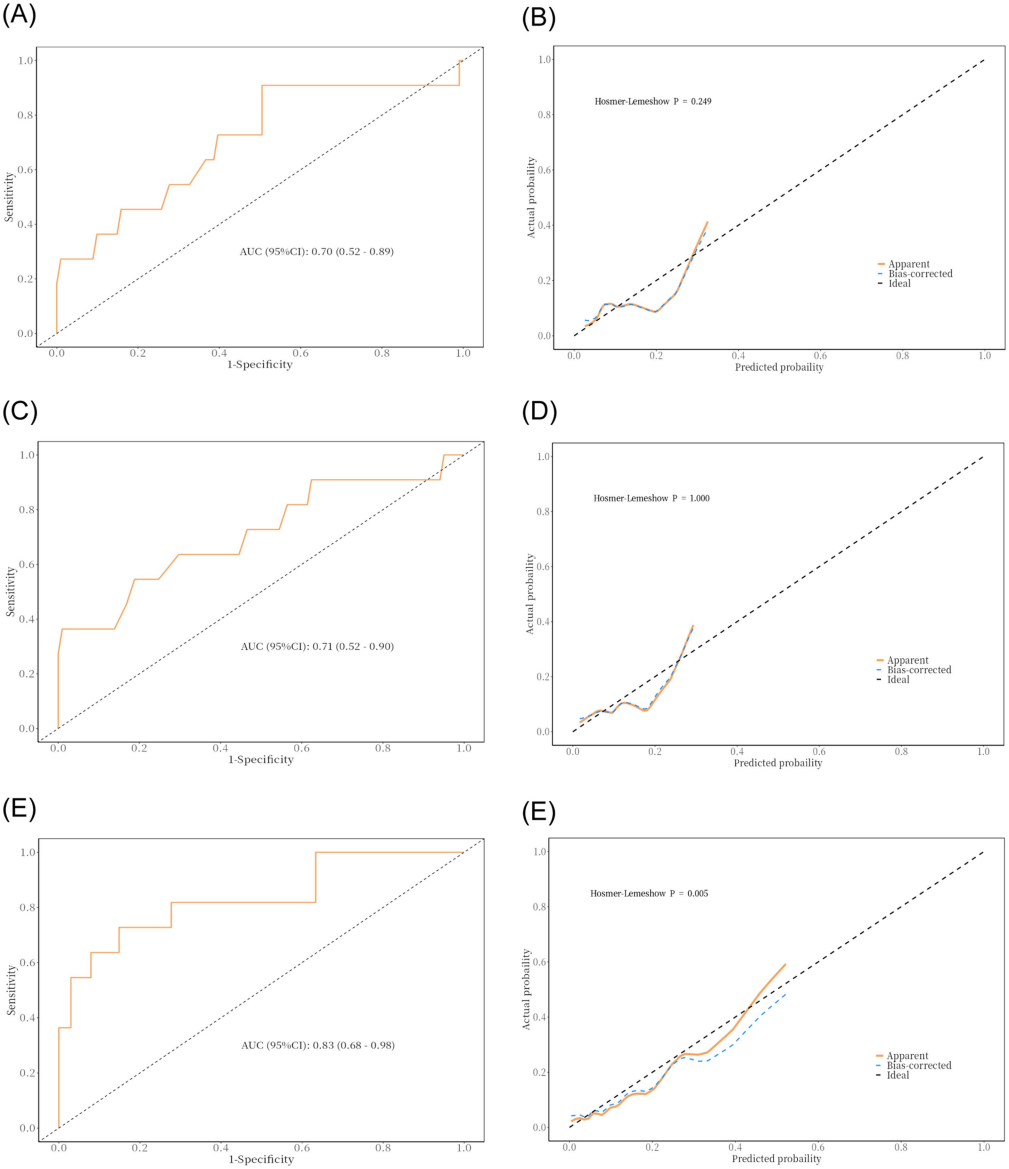
Result of ROC curve analysis and discriminability and calibration curves for the predictive model. **(A,B)** Area under the ROC curve of the Pre-HB and the Hosmer–Lemeshow test. **(C,D)** Area under the ROC curve of the Post-HB and the Hosmer–Lemeshow test. **(E,F)** Area under the ROC curve of the multiple factors and the Hosmer–Lemeshow test.

**Figure 4:**
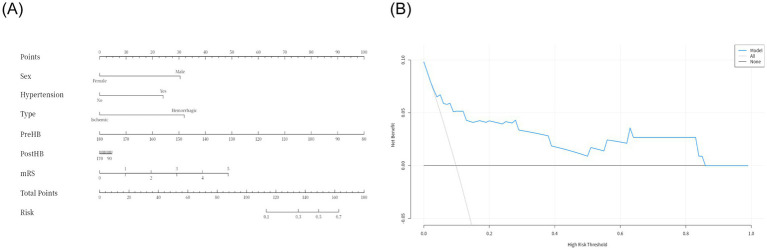
Visualization of the prediction model **(A)** and decision curve analysis **(B)**.

Due to delays in collecting data for Max HB, Min HB, and Mean HB, we included only Pre-HB and Post-HB in the ROC curve (Figure 3) to better apply the prediction model to the clinical study. The ROC curve analysis showed that the predictive performance of Pre-HB concentration for postoperative cerebral infarction was expressed as an area under the curve (AUC) = 0.70 (Figure 3A) (95%CI = 0.52–0.89), Youden index = 0.404, sensitivity = 49.5%, and specificity =90.9%. The optimal cutoff value for Pre-HB was 146.50 g/L. The predictive performance of Post-HB for postoperative cerebral infarction was expressed as area under the curve (AUC) = 0.71 (Figure 3C) (95%CI = 0.52–0.90), Youden index = 0.357, sensitivity = 81.2%, and specificity = 54.5%. The optimal cutoff value for Post-HB was 104 g/L. The Hosmer–Lemeshow test showed *p*-values of 0.249 and 1.000, respectively. However, the calibration curve between the prediction model and actual observations needs to be strengthened (Figures 3B,D). To further improve the performance of the prediction model and eliminate the influence of sex, we introduced high-risk factors (HTN, type of MMD, and mRS score) and sex into the ROC together with Pre-HB and Post-HB. The predictive performance of combined indicators for postoperative cerebral infarction was expressed as the area under the curve (AUC) = 0.83 (Figure 3E; 95%CI = 0.68–0.98), Youden index = 0.578, sensitivity = 72.7%, and specificity = 85.1%. The 1,000 bootstrap resampling strategy was used for optimistic correction, and the Hosmer–Lemeshow test showed a *p-*value of 0.005. The fit between the forecast model and the calibration curve was good (Figure 3F). The prediction model was visualized using a nomogram (Figure 4A). The decision curve analysis (DCA) (Figure 4B) suggested that a screening strategy based on nomograms of risk estimates for cerebral ischemic complications after MMD revascularization has a higher net benefit than “screen-none” or “screen-all” strategies if the threshold probability of individual is between 3 and 87%.

### 3.2 Association between HB and long-term prognosis of patients with moyamoya disease

All patients were followed up, and their historical medical records were reviewed. On this basis, the mRS scores of patients more than 1 year after surgery were recorded, and the patients were divided into two groups: group A (mRS = 0–2) and group B (mRS = 3–6). Subsequently, we performed a group difference analysis of the HB values between the two groups. The analysis results showed that the five types of HB values of patients with mRS scores of 0–2 were significantly higher than those with mRS scores of 3–6, and the differences were statistically significant (Table 2).

**Table 2:** Relationship between long-term prognosis and HB in patients with moyamoya disease.

Variables	Total (*n* = 112)	>1 year after surgery mRS score		*p*
		0–2 (*n* = 84)	3–6 (*n* = 28)	
Pre-HB, Mean ± SD	138.82 ± 19.19	141.20 ± 17.46	131.68 ± 22.52	0.022
Post-HB, Mean ± SD	119.40 ± 19.35	122.82 ± 17.33	109.14 ± 21.69	<0.001
Max HB, Mean ± SD	139.19 ± 19.28	141.85 ± 17.31	131.21 ± 22.78	0.011
Min HB, Mean ± SD	113.31 ± 19.44	116.43 ± 17.49	103.96 ± 22.19	0.003
Mean HB, Mean ± SD	124.61 ± 18.01	127.56 ± 15.75	115.75 ± 21.51	0.011

## 4 Discussion

MMD is considered a disease with a genetic basis, and studies have shown that its familial inheritance tendency is significant; approximately 10% of patients with MMD can be traced back to their family history (24). The inheritance pattern of familial moyamoya disease appears to follow an autosomal dominant pattern with incomplete penetrance, and the key gene locus may be located on chromosome 17q25.3 (25). Although revascularization is the most successful treatment for patients with MMD, it has a relatively high risk of postoperative cerebral infarction. Deng et al. reported that the probability of an ischemic event after 533 revascularizations was 8.9% per patient (26). Bao et al. reported a 4.8% incidence of postoperative transient ischemic attack or cerebral infarction per patient after 512 revascularizations (27). Kim et al. reported that after 845 revascularization procedures, the incidence of postoperative infarction was 13% per patient (28). Therefore, we attempted to study the relationship between HB and postoperative cerebral infarction in patients with MMD and to establish a relevant prediction model based on Hb levels.

Through statistical analysis, we found that low hemoglobin levels were a risk factor for postoperative cerebral infarction in patients with moyamoya disease. We then collected data on why low HB levels might cause postoperative cerebral infarction in patients with MMD by reviewing the literature. First, reperfusion after direct bypass leads to the production of large amounts of superoxide anions, which further damage damaged vessels (29). In turn, low HB also triggers elevated inflammatory markers, such as C-reactive protein, tumor necrosis factor-alpha (TNF-*α*), and certain interleukins (ILs), while it can upregulate inducible nitric oxide synthase and CXC chemokine receptors, all of which increase the risk of postoperative brain injury in patients after revascularization (30–33). Second, low HB prevents the body’s coagulation function because red blood cells themselves adhere to the damaged vessel wall to participate in hemostasis and mediate the radial transport of platelets to the damaged vessel wall, as well as the interaction with platelets and fibrinogen to form a blood clot contraction (16, 34, 35), which is disadvantageous for patients after revascularization. Third, according to the literature, low HB results in a relative “reduction” of region cerebral blood flow in specific brain regions, possibly due to chronic ischemia that fails to trigger a normal vasodilatory response (36), resulting in reduced metabolism in the region, which leads to cognitive dysfunction and executive dysfunction, especially executive dysfunction (37). It is unclear whether a normal and complete diastolic response exists in newly anastomosed vessels after revascularization. The relevant literature also indicates that: (1) direct competition between the recipient vessel and regional cerebral blood flow bypass may lead to postoperative bypass occlusion; and (2) the thickened STA may not match the diameter of the atrophied cerebral cortical arteries, resulting in lower blood flow in the reconstructed cerebral cortical arteries (38). These factors can further impede the compensatory increase in cerebral blood flow, making patients more likely to experience postoperative bypass occlusion and more susceptible to decreased blood flow and oxygen levels. Fourth, low HB levels impair cerebrovascular autoregulation (39, 40), leading to fluctuations in cerebral perfusion and altered oxygen transport to the brain. The risk of an unstable cerebral hemodynamic state exists in patients themselves after revascularization (41), and both increase the turbulent state of the blood to some extent (42). Simultaneously, the hyperdynamic circulation caused by anemia regulates the expression of adhesion molecules by upregulating vascular endothelial cell production. This triggers an inflammatory response (43). All of these factors greatly increase the risk of cerebrovascular thrombosis and dislodgement. In addition, animal studies provide evidence that anemic hypoxia may exacerbate neurological damage due to permanent focal ischemia (36, 44, 45). One study showed that when Hb concentrations dropped below 90 g/dL, the incidence of cerebral hypoxia and cellular energy dysfunction greatly increased (46). In recent years, it has been reported that erythropoietin improves outcomes in animal models of SAH (47) and reduces neuronal ischemia (48) and that erythropoietin and hemoglobin may interact (49); however, relevant studies are still scarce.

Furthermore, it has not been demonstrated whether low HB is a result or a cause of poor postoperative cerebral functional outcomes. Lower HB levels during hospitalization may be a marker of the poor physiological status of the patient at the time of hospitalization. Patients with severe postoperative illnesses are at risk of lower HB and require more frequent blood tests to monitor their condition and more intravenous fluids. Our study did not confirm a causal relationship between HB and cerebral ischemic complications after MMD revascularization. This will be the focus of future research. Red blood cell transfusion is effective in improving cerebral oxygen delivery (50). Although studies by Zygun et al. have shown that red blood cell transfusion improves cerebral tissue oxygenation, it does not have a significant effect on cerebral metabolism, as measured by the lactate-pus-ketoacid ratio (51). Research by Poyraz and Sheth suggested that concentrated red blood cell transfusions may reduce mortality from cerebral hemorrhage when the patient has a more serious disease, whereas no independent association was found between red blood cell transfusions and adverse outcomes from intracranial hemorrhage (50). However, it is unclear whether HB levels trigger red blood cell transfusion (52). In the Jennifer Diedler report, which included patients with traumatic brain injury and subarachnoid hemorrhage, the results showed that the transfusion of red blood cells is associated with a higher risk of cerebral malignant tumors and poor prognosis (44).

Studies have demonstrated the adverse effects of low HB levels on cardiovascular and cerebrovascular diseases; however, few studies have been conducted in the context of MMD. Due to the presence of multiple HB measurements during hospitalization, we divided the HB concentrations into preoperative, postoperative, mean, and minimum, and maximum values to minimize errors. Finally, we found that lower HB in patients with MMD undergoing revascularization was an independent risk factor for postoperative cerebral infarction. Compared to Mean HB, Max HB, and Min HB values, Pre-HB, and Post-HB values have the advantage of immediacy, as doctors can obtain these values through routine blood tests without delays from additional data collection. This makes Pre-HB and Post-HB values more timely and practical, enabling doctors to make informed clinical decisions. After obtaining these key data, we applied them to a carefully constructed predictive model. By comprehensively introducing the patient’s Pre-HB and Post-HB values and other key indicators, the model can accurately assess the risk of postoperative cerebral infarction to provide strong support for doctors to formulate individualized and accurate treatment plans before and after surgery. The results of this study are expected to provide new ideas and methods for the prevention of postoperative cerebral infarction. Several limitations should be acknowledged in our study: (1) There is a delay in collecting data for Max HB, Min HB, and Mean HB; (2) the study is retrospective and based on single-center data, which carries inherent limitations and potential bias; (3) due to the rarity of MMD and its regional distribution, the relatively small sample size limits further statistical analysis. Consequently, large-scale studies are needed to confirm these findings.

## 5 Conclusion

Low HB is an independent risk factor for postoperative cerebral infarction in patients with moyamoya disease and leads to a worse long-term prognosis. Based on the statistical results, we established a prediction model including Pre-HB, Post-HB, sex, type of MMD, and HTN, so that surgeons can effectively identify patients at a high risk of postoperative cerebral infarction.

## Data Availability

The original contributions presented in the study are included in the article/Supplementary material, further inquiries can be directed to the corresponding author/s.
